# C1GALT1 overexpression promotes the invasive behavior of colon cancer cells through modifying O-glycosylation of FGFR2

**DOI:** 10.18632/oncotarget.1815

**Published:** 2014-03-15

**Authors:** Ji-Shiang Hung, John Huang, Yo-Chuen Lin, Miao-Juei Huang, Po-Huang Lee, Hong-Shiee Lai, Jin-Tung Liang, Min-Chuan Huang

**Affiliations:** ^1^ Graduate Institute of Clinical Medicine, National Taiwan University, Taipei, Taiwan; ^2^ Graduate Institute of Anatomy and Cell Biology, National Taiwan University, Taipei, Taiwan; ^3^ Department of Surgery, National Taiwan University Hospital, Taipei, Taiwan; ^4^ Department of Medical Research, National Taiwan University Hospital, Taipei, Taiwan; ^5^ Research Center for Developmental Biology and Regenerative Medicine, National Taiwan University, Taipei, Taiwan

**Keywords:** glycosylation, receptor tyrosine kinase, colorectal cancer

## Abstract

Core 1 β1,3-galactosyltransferase (C1GALT1) transfers galactose (Gal) to N-acetylgalactosamine (GalNAc) to form Galβ1,3GalNAc (T antigen). Aberrant O-glycans, such as T antigen, are commonly found in colorectal cancer. However, the role of C1GALT1 in colorectal cancer remains unclear. Here we showed that C1GALT1 was frequently overexpressed in colorectal tumors and is associated with poor survival. C1GALT1 overexpression promoted cell survival, migration, invasion, and sphere formation as well as tumor growth and metastasis of colon cancer cells. Conversely, knockdown of C1GALT1 with small interference (si) RNA was sufficient to suppress these malignant phenotypes in vitro and in vivo. Moreover, we are the first to show that fibroblast growth factor receptor (FGFR) 2 carried O-glycans in colon cancer cells. Mechanistic investigations showed that C1GALT1 modified the O-glycans on FGFR2 and enhanced bFGF-triggered activation of FGFR2 as well as increased bFGF-mediated malignant phenotypes. In addition, BGJ398, a selective inhibitor of FGFR, blocked the effects of C1GALT1. These findings suggest that C1GALT1 overexpression modifies O-glycans on FGFR2 and enhances its phosphorylation to promote the invasive behavior and cancer stem-like property in colon cancer cells, indicating a critical role of O-glycosylation in the pathogenesis of colorectal cancer.

## INTRODUCTION

Colorectal cancer is the third most common malignant disease and the fourth leading cause of cancer-related death worldwide [1]. Because of limited treatment modalities and therapeutic agents, the prognosis of late-stage colorectal cancer remains dismal [2]. In the past decades, several important genetic factors and molecular pathways controlling colorectal cancer growth and metastasis have been elucidated [3]. But functional roles of the posttranslational modifications, such as glycosylation, in colorectal cancer still remain unclear. To improve the results of treating advanced colorectal cancer, further understanding the mechanisms controlling colorectal cancer progression and identifying targets for developing new therapeutic agents are still required.

Mucin-type *O-*glycosylation is one of the most common post-translational modifications of proteins in the gastrointestinal tract, initiated by the transfer of *N*-acetylgalactosamine (GalNAc) to serine or threonine residue forming Tn antigen [4]. This reaction is catalyzed by a large family of enzymes known as the UDP-GalNAc:polypeptide *N*-acetylgalactosaminyltransferases (GALNTs) [5]. Core 1 β1,3-galactosyltransferase (C1GALT1), with the help of its chaperone Cosmc, transfers galactose (Gal) to Tn antigen to form Galβ1,3GalNAc (T antigen) [6]. In normal tissues, T antigen is further modified by other glycosyltransferases to generate more complex *O*-glycans [7].

Mucin-type *O*-glycosylation regulates a wide range of biological functions. Death receptor *O*-glycosylation modulates apoptosis signaling in cancer cells [8]. Initiation of mucin-type *O*-glycosylation in the endoplasmic reticulum promotes cancer cell invasiveness [9]. Mice lacking core 1-derived *O*-glycans exhibit defective angiogenesis and fatal embryonic hemorrhage [10]. C1galt1-deficient mice showed abnormal differentiation of megakaryocytes [11]. Loss of intestinal core 1-derived *O*-glycans causes spontaneous colitis in mice [12]. A recent report suggested that hypoglycosylated MUC1 modulates breast cancer cell migration and invasion [13]. Moreover, we recently showed that C1GALT1 modifies *O*-glycans on HGF receptor (MET) to enhance cell proliferation in liver cancer cells [14]. These results strongly suggest relevant roles of *O*-glycosylation in tissue development and diseases.

Aberrant *O*-glycosylation has been suggested to play critical roles in tumor malignancy and is associated with survival of cancer patients [15-19]. T antigen is commonly found in colon, breast, ovarian, and prostate cancers [20, 21] but not in their normal counterparts and has been suggested to regulate cancer metastasis, proliferation, and angiogenesis [13, 22-24]. Although it is widely accepted that *O*-glycans are highly expressed in colorectal tissues and changes in *O*-glycans may play significant roles in tumor progression, the expression and function of C1GALT1, a key enzyme for *O*-glycan biosynthesis, in colorectal cancer remain unknown.

## RESULTS

### C1GALT1 is overexpressed in colorectal tumors and associated with poor survival

**Figure 1:**
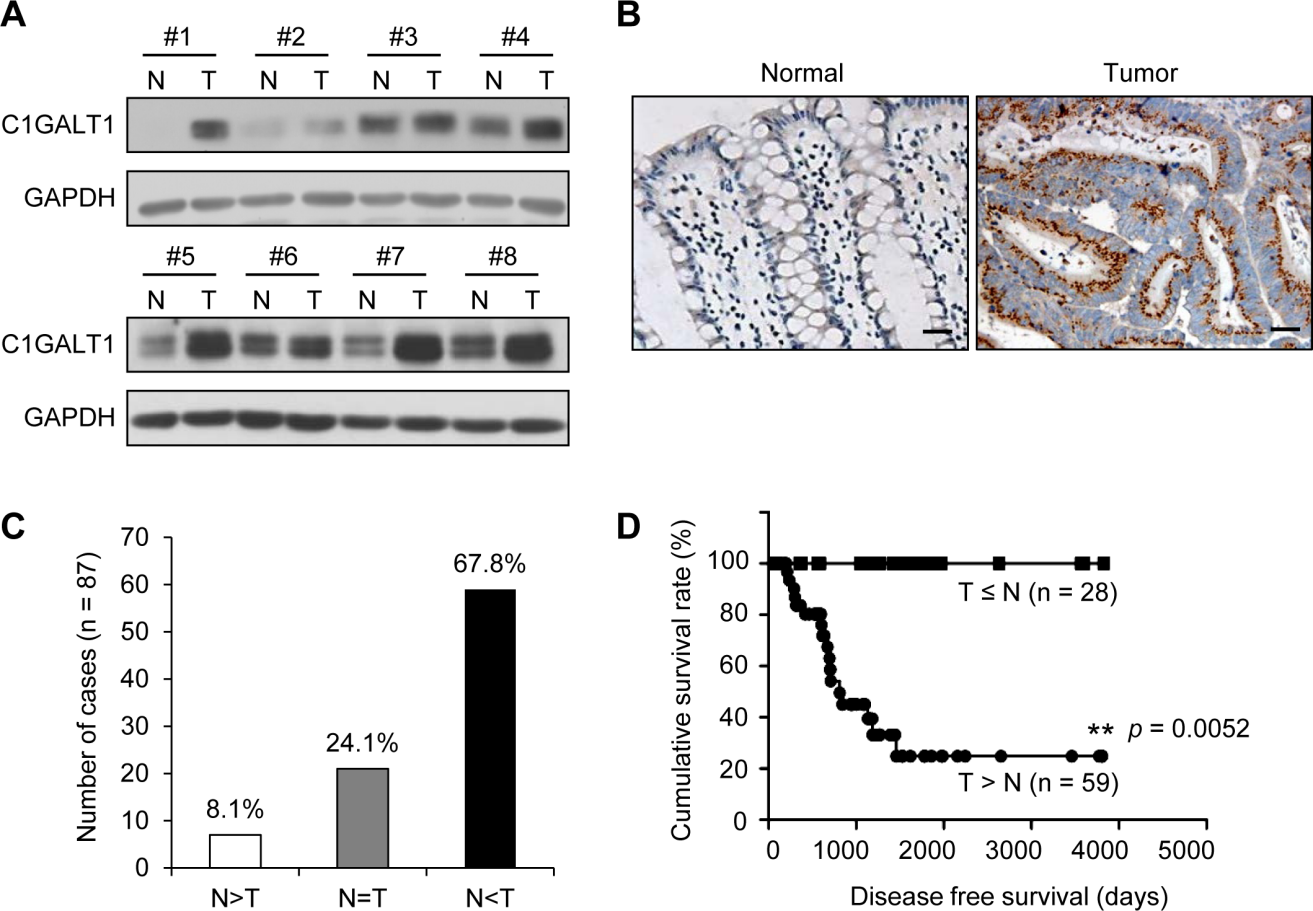
C1GALT1 is frequently overexpressed in colorectal tumors and its overexpression predicts poor survival of colorectal cancer patients (A) Western blots of C1GALT1 expression in paired normal (N) and tumor (T) tissue from colorectal cancer patients (n = 8). (B) Immunohistochemistry (IHC) of C1GALT1 expression in paired normal and tumor tissue of colorectal cancer. Representative images are shown. Negative controls did not show any specific signals (data not shown). Scale bar indicates 25 μm. (C) C1GALT1 is frequently overexpressed in colorectal tumors (T) compared with their adjacent normal tissues (N). (D) C1GALT1 overexpression predicts poorer survival of patients with colorectal cancer. Cumulative survival rate was analyzed by Kaplan-Meier survival analysis for colorectal cancer patients (n = 87). **p < 0.01.

To determine the expression of C1GALT1 in human colorectal tissues, paired tissues (n = 8) from patients of the National Taiwan University Hospital (NTUH) were analyzed by Western blotting (Figure 1A). C1GALT1 expression was found to be overexpressed in colorectal cancer tissues compared with adjacent non-tumor tissues. Immunohistochemical analysis confirmed that C1GALT1 was up-regulated in colorectal adenocarcinoma cells compared with adjacent normal epithelial cells (Figure 1B). The results from immunohistochemistry of C1GALT1 indicated that 67.8% (59/87) of colorectal tumors showed higher C1GALT1 expression than their normal counterpart tissues (Figure 1C). Moreover, a Kaplan-Meier survival analysis showed that the cumulative survival rate of colorectal cancer patients with C1GALT1 overexpression (T > N) was significantly lower than the patients without C1GALT1 overexpression (T ≤ N) (Figure 1D). In addition, we also observed that C1GALT1 expression is positively associated with T antigen expression as revealed by PNA staining (Supplementary Figure S1). These findings suggest that C1GALT1 expression is frequently overexpressed in colorectal tumors and its overexpression is associated with poor survival.

### C1GALT1 regulates malignant phenotypes and stem-like properties of colon cancer cells

**Figure 2:**
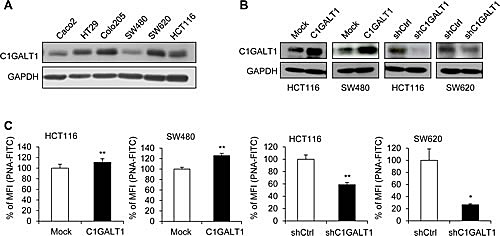
C1GALT1 expression in colon cancer cells (A) Expression of C1GALT1 in six colon cell lines was analyzed by Western blotting. GAPDH is an internal control. (B) Western blots showing overexpression and knockdown of C1GALT1 in colon cancer cells. C1GALT1 was stably overexpressed by transfection with empty vector (Mock) or *C1GALT1*/pcDNA3.1 plasmid (C1GALT1) in HCT116 and SW480 cells. C1GALT1 was stably knocked down with control shRNA (shCtrl) or C1GALT1-specific shRNA (shC1GALT1) in HCT116 and SW620 cells. (C) Effects of C1GALT1 on T antigen expression of colon cancer cell surfaces. Surface T antigens were detected by flow cytometry with FITC-PNA and results are presented as percent mean fluorescence intensity (% of MFI) compared with mock, normalized to cell number and background fluorescence, from three independent experiments. *p < 0.05; **p < 0.01.

To investigate roles of C1GLAT1 in colon cancer cells, we first analyzed C1GALT1 expression in six colon cancer cell lines Caco2, HT29, Colo205, SW480, SW620, and HCT116 by Western blotting. C1GALT1 was expressed in colon cancer cells at different levels (Figure 2A). Low metastatic SW480 cell line was isolated from the primary colon tumor, and the high metastatic SW620 cell line is isolated from the lymph node of the same patient. These two cell lines are often used to study the mechanism of colon cancer metastasis. Interestingly, the expression level of C1GALT1 is higher in SW620 cells than SW480 cells, which is in agreement with our hypothesis that C1GALT1 may enhance malignant behaviors of colorectal cancer. We therefore selected SW480 cells for overexpression and SW620 cells for knockdown of C1GALT1. In addition, we overexpressed and knocked down C1GALT1 in HCT116 cells, which express C1GALT1 at a moderate level, to analyze effects of C1GALT1. The stable overexpression and shRNA-mediated knockdown of C1GALT1 in colon cancer cells were confirmed by Western blotting (Figure 2B). Moreover, flow cytometry with PNA lectin showed that C1GALT1 overexpression enhanced T antigen expression, whereas C1GALT1 knockdown inhibited T antigen expression (Figure 2C).

**Figure 3:**
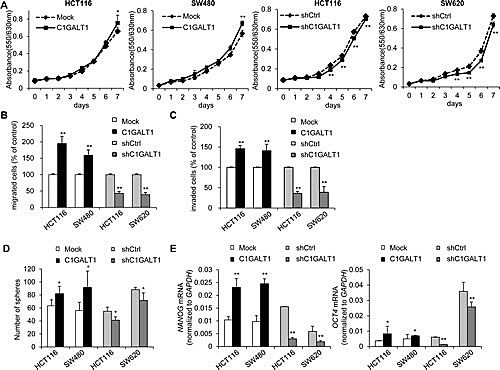
C1GALT1 regulates malignant phenotypes of colon cancer cells (A) Effects of C1GALT1 on viability of colon cancer cells. Cell viability was analyzed in C1GALT1 overexpressing HCT116 and SW480 cells and in C1GALT1 knockdown HCT116 and SW620 cells by MTT assays. **p < 0.01. (B) Effects of C1GALT1 on cell migration. Cell migration was analyzed by transwell migration assays. DMEM containing 10% FBS were used as chemoattractants. After 48 h, the number of migrated cells from 6 random fields was counted. Results are presented as mean ± SD from three independent experiments. **p < 0.01. (C) Effects of C1GALT1 on cell invasion. Cell invasion was analyzed by matrigel invasion assays. **p < 0.01. Similar analyses were used as those for migration assays. (D) Effects of C1GALT1 on sphere formation. Sphere formation assays were performed in serum free medium supplemented with B27 and treated with 20 μg/ml EGF and 25 μg/ml bFGF. The number of spheres formed was counted after visualizing by MTT. Results are presented as mean ± SD from three independent experiments. *p < 0.05. (E) Effects of C1GALT1 on the expression of stem cell markers. C1GALT1 modulated the mRNA expression of *NANOG* and *OCT4* analyzed by real-time RT-PCR. *GAPDH* is an internal control. *p < 0.05; **p < 0.01.

To investigate effects of C1GALT1 on malignant phenotypes, we analyzed cell viability, migration and invasion in colon cancer cells. Results from MTT assay showed that overexpression of C1GALT1 slightly increased cell viability in HCT116 and SW480 cells, whereas knockdown of C1GALT1 slightly inhibited cell viability in HCT116 and SW620 cells (Figure 3A). We next analyzed migration and invasion by transwell and matrigel invasion assay, respectively. Results showed that overexpression of C1GALT1 significantly enhanced cell migration and invasion in HCT116 and SW480 cells (Figure 3B & 3C). The images of migrated and invaded cells were shown in Supplementary Figure S2. In contrast, knockdown of C1GALT1 suppressed cell migration and invasion in HCT116 and SW620 cells (Figure 3B & 3C; Supplementary Figure S2). Moreover, transient knockdown of C1GALT1 with two different siRNAs confirmed the role of C1GALT1 in migration and invasion of colon cancer cells (Supplementary Figure S3). These findings suggest that C1GALT1 can regulate malignant behaviors of colon cancer cells.

Since cancer stem cells play a critical role in malignant diseases, we therefore investigated whether C1GALT1 is able to modulate the stem-like properties in colon cancer cells. Sphere formation assays were performed to measure the self-renewal ability, where cells were maintained in serum free medium supplemented with 20 μg/ml EGF and 25 μg/ml bFGF. Our results showed that overexpression of C1GALT1 significantly increased sphere forming ability in HCT116 and SW480 cells (Figure 3D). Conversely, knockdown of C1GALT1 reduced the number of spheres in HCT116 and SW620 cells. Furthermore, we also analyzed the expression of stem cell associated markers, *NANOG* and *OCT4*, by real-time RT-PCR. Both *NANOG* and *OCT4* mRNA were increased in C1GALT1 overexpressing HCT116 and SW480 cells, whereas they were decreased in C1GALT1 knockdown HCT116 and SW620 cells (Figure 3E). These findings suggest that overexpression of C1GALT1 enhances stem-like properties of colon cancer cells.

### bFGF signaling pathways are involved in the phenotypic changes mediated by C1GALT1

**Figure 4:**
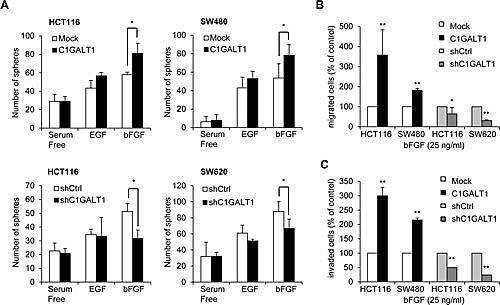
C1GALT1 regulates bFGF-induced malignant phenotypes in colon cancer cells (A) Effects of C1GALT1 on sphere formation induced by EGF or bFGF in colon cancer cells. Sphere formation assays were performed in serum free medium supplemented with 1 × B27 and treated with 20 μg/ml EGF or 25 μg/ml bFGF. Numbers of spheres formed were counted. Results are presented as mean ± SD from three independent experiments. *p < 0.05. (B) Effects of C1GALT1 on bFGF-mediated cell migration. For transwell migration assays, 25 μg/ml bFGF in serum free DMEM was used as chemoattractants. Results are displayed as percentage of migrated cells relative to control and presented as mean ± SD from three independent experiments. *p < 0.05. **p < 0.01. (C) Effects of C1GALT1 on bFGF-induced cell invasion analyzed by matrigel invasion assays **p < 0.01.

We next investigated the mechanisms by which C1GALT1 regulates malignant phenotypes of colon cancer cells. Since EGF and bFGF play critical roles in malignant progression and stemness in many cancers and we have found that C1GALT1 can modulate sphere forming ability induced by EGF and bFGF, we therefore analyzed whether these two signaling pathways are involved in the C1GALT1-mediated phenotypic changes. We found that overexpression of C1GALT1 significantly enhanced sphere formation induced by bFGF, but not EGF, in HCT116 and SW480 cells (Figure 4A). In contrast, knockdown of C1GALT1 significantly decreased sphere formation induced by bFGF, but not EGF, in HCT116 and SW620 cells. Furthermore, overexpression of C1GALT1 promoted bFGF-induced migration (Figure 4B) and invasion (Figure 4C), whereas knockdown of C1GALT1 inhibited bFGF-induced migration and invasion (Figure 4B and 4C).

In addition, BGJ398, a potent and selective inhibitor for FGFRs, significantly blocked the effects of C1GALT1 on cell migration, invasion, and sphere formation in colon cancer cells (Supplementary Figure S4). BGJ398 could also inhibit the effects of C1GALT1 on bFGF-triggered cell migration and invasion (Supplementary Figure S5). The inhibitory effect of BGJ398 on FGFR activity was confirmed by decreased phosphorylation of ERK1/2 (Supplementary Figure S6). Collectively, these results indicate that bFGF signaling pathways are involved in cancer stemness and malignant phenotypes induced by C1GALT1 in colon cancer cells.

### C1GALT1 expression modulates tumor growth and tumor metastasis in immunodeficient mouse models

**Figure 5:**
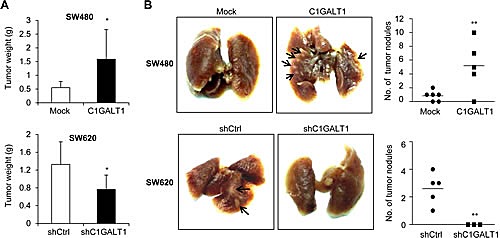
C1GALT1 regulates tumor metastasis and growth in NOD/SCID mice (A) Effects of C1GALT1 on tumor growth. C1GALT1 overexpressing SW480 or C1GALT1 knockdown SW620 cells were injected subcutaneously (n = 4 for SW620 shC1GALT1; n = 5 for other groups). Tumor were excised after 6 weeks and weighted. Data are presented as mean ± SD. *p < 0.05. (B) Effects of C1GALT1 on metastasis in a mouse rectal xenograft model. Representative images of lung metastasis are shown and total numbers of metastatic tumor nodules on surfaces of lungs are counted. C1GALT1 was overexpressed in SW480 cells (upper panels). C1GALT1 was knocked known with shRNA in SW620 cells (lower panels). Arrows indicate metastatic tumor nodules. **p < 0.01.

To investigate the effect of C1GALT1 on tumor growth *in vivo,* we performed subcutaneous injection of colon cancer cells in NOD/SCID mice. Results showed that overexpression of C1GALT1 in SW480 cells increased tumor weights, whereas knockdown of C1GALT1 in SW620 cells decreased tumor weights (Figure 5A). In addition, the effect of C1GALT1 on tumor metastasis *in vivo* was evaluated by rectal xenograft model. Colon cancer cells were submucosally injected into the rectum of NOD/SCID mice. We observed that overexpression of C1GALT1 increased lung metastasis of SW480 cells, whereas knockdown of C1GALT1 suppressed lung metastasis of SW620 cells (Figure 5B). These results suggest that C1GALT1 expression is able to modulate tumor growth and metastasis *in vivo*.

### C1GALT1 modifies FGFR2 glycosylation and activity in colon cancer cells

**Figure 6:**
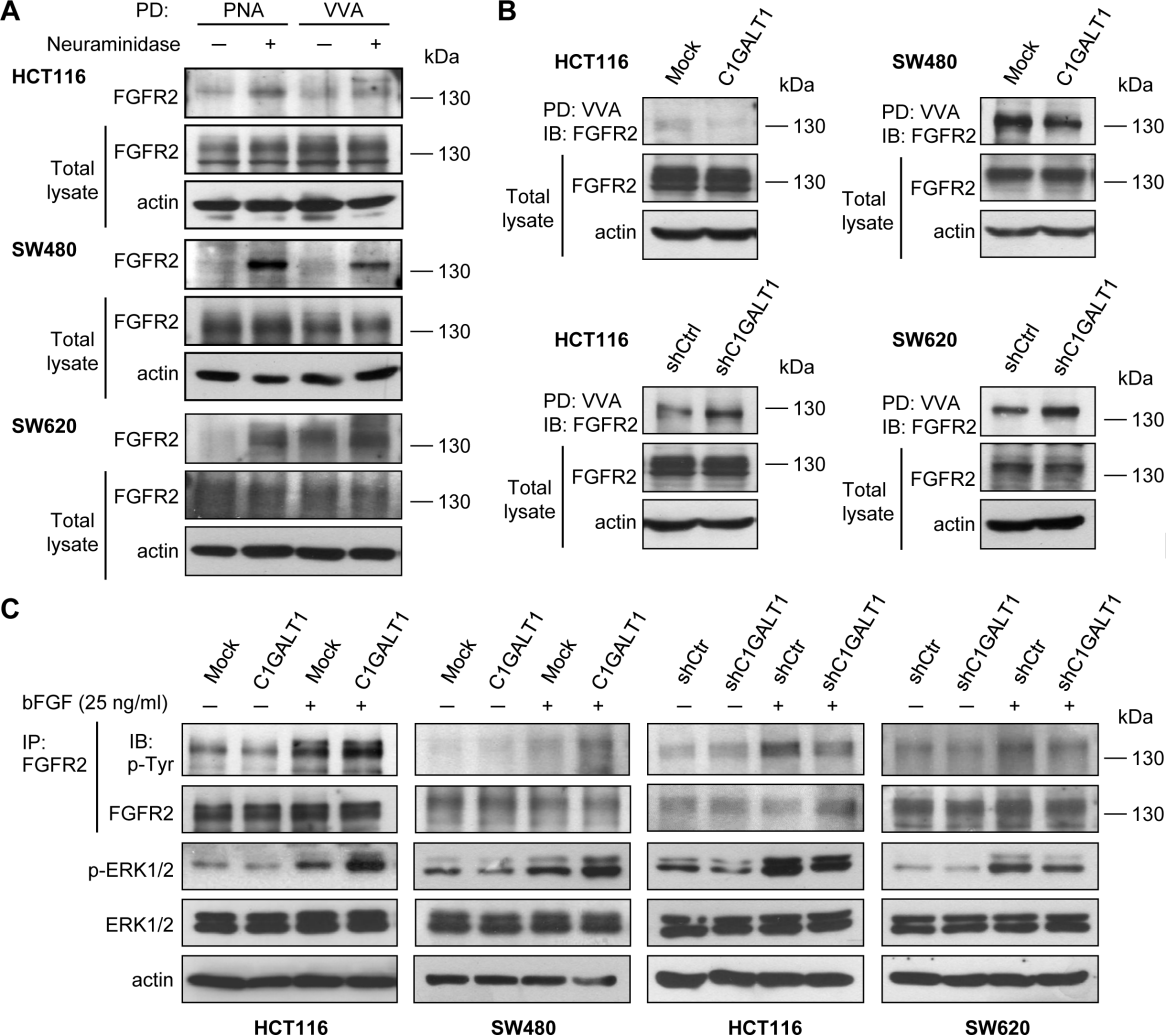
C1GALT1 modifies O-glycans on FGFR2 and regulates bFGF-induced FGFR2 activation in colon cancer cells (A) FGFR2 carries *O*-glycans in colon cancer cells. Lysates of HCT116, SW480, and SW620 cells were treated with (+) or without (-) neuraminidase and pulled down (PD) by either PNA or VVA lectin agarose beads and blotted with anti-FGFR2 antibody. Actin is an internal control. (B) C1GALT1 modifies FGFR2 *O*-glycosylation in colon cancer cells. Lysates from C1GALT1 overexpressing HCT116 and SW480 cells (upper panels) and C1GALT1 knockdown HCT116 and SW620 cells (lower panels) were subjected to VVA lectin pull down (PD) and immunoblotted with anti-FGFR2 antibody. Actin is an internal control. (C) C1GALT1 expression regulated FGF-induced phosphorylation of FGFR2 and ERK1/2. Cells were serum starved and treated with (+) or without (-) bFGF 25 ng/ml for 5 min. Cell lysates were then immunoprecipitated with anti-FGFR2 antibody and blotted with anti-phosphotyrosine (4G10) or anti-FGFR2 antibodies. Levels of p-ERK1/2 and total ERK1/2 were also analyzed. Actin was used as a loading control.

We found that C1GALT1 can regulate bFGF-induced malignant phenotypes. In addition, among the four FGFRs (FGFR1-4), the significance of FGFR2 in colorectal cancer has been clearly demonstrated [25]. We therefore investigated whether C1GALT1 can regulate FGFR2 glycosylation and activity. We found that only low amounts of endogenous FGFR2 were pulled down by PNA in HCT116, SW480, and SW620 cells (Figure 6A), indicating small amounts of T antigens on FGFR2. Interestingly, after neuraminidase treatment, FGFR2 was easily pulled down by PNA, suggesting that FGFR2 carries sialyl T antigens. Moreover, we found that FGFR2 could also be pulled down by VVA, indicating the presence of Tn on FGFR2 (Figure 6A). Removal of sialic acids with neuraminidase increased VVA binding to FGFR2, indicating the presence of sialyl Tn on FGFR2. These results strongly suggest that FGFR2 is decorated with *O*-glycans in colon cancer cells.

We next investigated whether C1GALT1 can modify *O*-glycans on FGFR2. Our data showed that overexpression of C1GALT1 decreased VVA binding to FGFR2 in HCT116 and SW480 cells, whereas knockdown of C1GALT1 increased VVA binding to FGFR2 in HCT116 and SW620 cells (Figure 6B). These results suggest that C1GALT1 is able to modulate *O*-glycan structures on FGFR2 in colon cancer cells. Furthermore, we found that overexpression of C1GALT1 increased bFGF-induced phosphorylation of FGFR2 and ERK1/2 in HCT116 and SW480 cells (Figure 6C). Conversely, knockdown of C1GALT1 suppressed the phosphorylation of FGFR2 and ERK1/2 after bFGF treatment in HCT116 and SW620 cells (Figure 6C). These findings suggest that C1GALT1 modifies *O*-glycans on FGFR2 and regulates bFGF-induced activation of FGFR2 in colon cancer cells.

## DISCUSSION

FGFRs consist of four members, termed FGFR1, 2, 3, and 4 [25]. FGFs are ligands for FGFRs and the FGF/FGFR family plays crucial roles in carcinogenesis and tumor progression in many cancers [25]. All four FGFRs have been reported to be expressed in colorectal cancer. Among them, FGFR2 and its isoform are highly expressed in colorectal cancer and correlate with tumor growth, metastasis, and angiogenesis [25]. Several lines of evidence showed the effectiveness of targeting FGFR2 in colorectal cancer [26]. Our data showed that C1GALT1 can regulate *O*-glycosylation and phosphorylation of FGFR2 and its downstream signaling molecule ERK. Furthermore, BGJ398 significantly blocked the effects of C1GALT1 on the malignant behavior of colon cancer cells. These findings further support a critical role of C1GALT1 in colorectal cancer and suggest that FGF/FGFR2 signaling pathways are involved in C1GALT1-mediated phenotypic changes. Several glycoproteins in colorectal tissues are decorated with *O*-glycans. Therefore, it is reasonable to speculate that other molecules could cooperate with FGFR2 to mediate the effects of C1GALT1. Identifying other acceptor substrates of C1GALT1 by glycoproteomics will be of great help to unravel the detailed mechanisms by which C1GALT1 regulates cancer behaviors.

There is increasing evidence that the capacity of tumor growth resides in a small subpopulation of cells, called cancer-initiating cells or cancer stem-like cells [27]. These cells are more resistance to chemotherapy and radiotherapy in many cancers [28]. Eliminating cancer stem-like cells is therefore suggested to be essential for efficient therapies. We found that inhibiting C1GALT1 expression was able to reduce sphere-forming ability of colon cancer cells, suggesting that targeting C1GALT1 may be a promising strategy to decrease cancer stem-like cells in colorectal cancer.

It has been demonstrated that FGFR2 is *N*-glycosylated and its *N*-glycans can modulate FGFR2 activation and intracellular trafficking [29]. In this study, we found that FGFR2 could be pulled down by both VVA and PNA lectins and removal of sialic acids enhanced their binding to FGFR2, suggesting that FGFR2 carries short *O*-glycans, such as Tn, sialyl Tn, T, and sialyl T antigens in colon cancer cells. We also showed that C1GALT1 expression could affect VVA binding to FGFR2 and regulate FGFR2 phosphorylation, further demonstrating that FGFR2 is *O*-glycosylated. In support of our finding, the NetOGlyc 4.0 Server [30] predicts four potential O-glycosylation sites in the extracellular domain of FGFR2. These results for the first time show that FGFR2 is *O*-glycosylated and that the *O*-glycosylation can modulate FGFR2 activity in colon cancer cells.

Short *O*-glycans, such as T antigens, are commonly found in colorectal tumors and these carbohydrates have been associated with tumor progression and developed as vaccines for cancer treatments [31]. However, factors that determine the T antigen expression remain unclear. In colorectal cancer, downregulation of core 2 synthase [32] or core 3 synthase [33] has been suggested to increase T antigen expression. We recently found that Cosmc, a C1GALT1-specific chaperone, is able to enhance T antigen expression [34]. However, Cosmc is downexpressed in most colorectal tumors with low stages (stage I and II). In this study, we observed that both C1GALT1 and PNA-staining T antigen are frequently overexpressed in colorectal tumors at all stages. In addition, statistical analyses showed that C1GALT1 expression is positively associated with T antigen expression. These results suggest that C1GALT1 is a critical positive regulator of T antigen expression in colorectal cancer, which may coordinately control T antigen expression with negative regulators, such as core 2 synthase and core 3 synthase, *in vivo*. Notably, there is no significant correlation between Cosmc and C1GALT1 expression in colorectal tumors (data not shown), implying that, in addition to Cosmc, other factors are involved in the regulation of C1GALT1 expression.

In conclusion, we found that C1GALT1 is overexpressed in colorectal tumors and its overexpression is associated with poor survival of patients with colorectal tumors. C1GALT1 overexpression enhances the invasive potential and stem-like cell property of colon cancer cells via modifying *O*-glycosylation and activity of FGFR2. Conversely, C1GALT1 knockdown suppresses these malignant properties *in vitro* and *in vivo*. These findings open novel insights into the relevant role of *O*-glycosylation in colorectal cancer and suggest C1GALT1 as a promising therapeutic target for the treatment of colorectal cancer.

## MATERIALS AND METHODS

### Tissue samples

Human colorectal tissues were obtained from the Departments of Surgery and Pathology, National Taiwan University Hospital. The use of human tissues for this study was approved by the local hospital ethics committee, and written consent was obtained from patients before the collection of samples. Post-surgery human colorectal cancer tissues were collected for RNA extraction, western blotting and paraffin-embedded tissue sections. The clinicopathological data of the patients was provided in Supplementary Table S1.

### Western blotting

Equal amounts of protein samples were mixed with 5× sample buffer and boiled for 5 min, separated on SDS-polyacrylamide gels, and then transferred to PVDF membrane. The membranes were blocked in 5% BSA for 1 h at room temperature, and incubated with primary antibodies overnight at 4°C. Anti-phosphotyrosine (4G10) antibody (Millipore, Billerica, MA), antibodies against C1GALT1, GAPDH, FGFR2 (Santa Cruz Biotechnology, Santa Cruz, CA), anti-p-ERK1/2, anti-ERK1/2 andibodies (Cell Signaling Technology, Danvers, MA) and anti-β-actin antibody (BD Pharmingen, San Jose, CA) were used. The blots were then incubated with secondary antibody conjugated with horseradish peroxidase and immunoreacted bands were detected by ECL reagents and exposed on x-ray film.

### Immunohistochemistry

Paraffin-embedded tissue sections were incubated with an anti-C1GALT1 (1:300) antibody overnight at 4°C. Super Sensitive link-Label IHC detection System (BioGenex, California, CA) was used and signals were visualized with 3,3-diaminobenzidine (DAB) liquid substrate system (Sigma, St. Louis, MO). All sections were counterstained with hematoxylin. Negative controls were performed by replacing primary antibody with a control IgG at the same concentration. The staining intensity and positive ratio of C1GALT1 were observed under microscope by two independent scorers blinded to the clinical parameters.

### Cell culture and transfection

Human colon cancer cell lines HCT116, SW480, SW620, Caco2, HT29, Colo205 were purchased from Bioresource Collection and Research Center (Hsinchu, Taiwan). Cells were maintained with Dulbecco's modified Eagle's medium (Thermo scientific, Barrington, IL) containing 10% fetal bovine serum (GIBCO, Grand Island, NY), 100 IU/ml penicillin, and 100 mg/ml streptomycin in a humidified tissue culture incubator at 37°C, 5% CO2. For C1GALT1 overexpression, HCT116 and SW480 cells were transfected with C1GALT1/pcDNA3.1/mycHis plasmids using Lipofectamine 2000 (Invitrogen, Life Technologies Inc., Grand Island, NY) according to the manufacturer's protocol. Empty pcDNA3.1/mycHis plasmids were used as mock transfection. Stable clones were selected with 400 μg/ml of G418 for 14 days. For C1GALT1 knockdown, HCT116 and SW620 cells were transfected with short hairpin (sh) RNA and selected with 2 μg/ml of puromycin for 14 days. The pLKO/C1GALT1-shRNA plasmid and non-targeting pLKO plasmids were purchased from National RNAi Core Facility (Academia Sinica, Taipei, Taiwan). The overexpression and knockdown of C1GALT1 were confirmed by western blotting.

### cDNA synthesis and quantitative real-time RT-PCR

The total RNA was extracted by Trizol reagent (Invitrogen) according to the manufacturer's protocol. Extracted RNA was then reverse transcribed using the Superscript III First-Strand cDNA Synthesis Kit (Invitrogen). Quantitative PCR System Mx3000P (Stratagene, La Jolla, CA) was used for quantitative real-time PCR. Reactions were performed in a 20 μl volume with 2 μl cDNA, 400 nM of sense and anti-sense primers, and 10 μl Brilliant®SYBR®Green QPCR Master Mix (Stratagene). Primer sets were designed as the following:

*NANOG*, sense (5'-GGCCTCAGCACCTACCTACCC-3') and anti-sense (5'-TCCAAGGCAGCCTCCAAGTCA-3');

*OCT4*, sense (5'-GCAGATCAGCCACATCGCCC-3') and anti-sense (5'-GCCCAGAGTGGTGACGGAGA-3')

*GAPDH*, sense (5'-ACAGTCAGCCGCATCTTCTT-3') and anti-sense (5'-GACAAGCTTCCCGTTCTCAG-3')

Relative quantity of gene expression normalized to GAPDH was analyzed using MxPro Software (Stratagene).

### Cell viability assay

Cells (2.0×10^3^) were seeded in 96-well plates. After culture for different time periods, the 3-(4,5 dimethyl-2 thiazolyl)-2,5 diphenyl-2H tetrazolium bromide solution (MTT; Sigma) was added to a final concentration of 0.5 mg/ml and incubated for 4 h at 37°C to allow MTT reduction. The formazan crystals were then dissolved in 10% sodium dodecyl sulfate (SDS) containing 0.01 M HCl and absorbance was measured at the dual wavelengths of 570 and 630 nm with a spectrophotometer.

### Transwell migration assay

Cells (8×10^4^) in 500 μL DMEM containing 1% FBS were seeded into the upper part of the Boyden chamber with 8-μm pore filters (Corning, Cambridge, MA). Cell migration was induced by 10% FBS (GIBCO) or 25 ng/ml bFGF (Sigma) in the lower part. In some experiments, 1 μM BGJ398 (Santa Cruz Biotechnology) or 0.1% DMSO was included in the upper-chamber medium. After 48 h, cells that had migrated to the lower surface of the membrane were fixed and stained with 0.5% crystal violet (Sigma) and counted under a microscope in six random fields.

### Matrigel invasion assay

Cell invasion assays were performed in BioCoat Matrigel Invasion Chambers (Becton-Dickinson, Bedford, MA) according to the manufacturer's protocol. Briefly, 500 μl DMEM containing 10% FBS (GIBCO) or 25 ng/ml bFGF (Sigma) were added to the lower part of the chamber, whereas cells (8×10^4^) in 500 μL DMEM were seeded to the upper part. Cells were allowed to invade the matrigel for 48 h. In some experiments, 1 μM BGJ398 (Santa Cruz Biotechnology) or 0.1% DMSO was included in the upper-chamber medium. Cells that invaded to the lower surface of the membrane were fixed and stained with 0.5% crystal violet (Sigma) and counted under a microscope in six random fields.

### Flow cytometry

Detached cells were incubated with FITC-conjugated PNA (Merck Bioscience) on ice for 30 min to stain cell surface expression of T antigen. Cells were than analyzed with flow cytometry (BD Bioscience). Results were present as mean fluorescence intensity.

### Sphere formation assay

Cells were suspended in DMEM/F12 (1:1) supplemented with B27 supplement (Invitrigen), epidermal growth factor (EGF) and recombinant fibroblast growth factor basic (bFGF) (Sigma). EGF (20 ng/ml) and bFGF (25 ng/ml) were used together or separately depending on experimental designs. Cells were seeded in Ultra-Low attachment 24-well plates (Corning) at a density of 1,000 cells per well. In some experiments, 1 μM BGJ398 (Santa Cruz Biotechnology) or 0.1% DMSO was added. After 10-14 days, MTT solution (Sigma) was added to visualize any spheres formed and pictures were taken. The number of spheres was quantified by ImageJ 1.42q software (Wayne Rasband).

### Lectin pull down and immunoprecipitation

To detect the T, Tn, sialyl T and sialyl Tn antigens on FGFR2, peanut agglutinin (PNA) and *Vicia villosa* agglutinin (VVA) lectins conjugated agarose beads (Vector Laboratories, Burlingame, CA) were used; neuraminidase was used to remove sialic acids. Briefly, 1-2 mg of cell lysates were treated with or without neuraminidase at 37°C for 1 h and incubated with PNA or VVA overnight at 4°C. For immunoprecipitation, cell lysates were incubated with 2.5 μg anti-FGFR2 antibody overnight at 4°C. Next, protein G sepharose beads (GE Healthcare Life Sciences, Piscataway, NJ) were added and incubated at 4°C for 4 h. The precipitated agarose beads were washed several times with lysis buffer to remove any unbound protein and then subjected to western blotting.

### In vivo metastasis model

For the rectal xenograft model, stable cell lines (2×10^6^ cells in 100 μl PBS) were submucosally injected into the rectum of 6-week-old female NOD/SCID mice (National Laboratory Animal Center, Taiwan) at day 0. The health status was monitored. After 6 weeks, mice were sacrificed and inspected for any tumor formed. Animal experiments were reviewed and approved by the Institutional Animal Care and Use Committee (IACUC) of National Taiwan University College of Medicine.

### In vivo tumor growth model

For tumor growth analysis, 6-week-old female NOD-SCID mice (National Laboratory Animal Center, Taiwan) were injected subcutaneously with 1×10^7^ of cells. Tumors were allowed to develop for 6 weeks. At day 42 after injection, tumors in each group were excised for analyses. Animal experiments were reviewed and approved by the Institutional Animal Care and Use Committee (IACUC) of National Taiwan University College of Medicine.

### Statistical analyses

Quantitative data from at least three independent experiments are expressed as means ± standard deviation (SD). Student's *t*-tests were used to compare the differences between groups. Kaplan-Meier analysis and the *log-rank* test were used to estimate overall survival. *P* < 0.05 is considered statistically significant.

## SUPPLEMENTARY FIGURES AND TABLES




