# Supplementary material for: C1GALT1 overexpression promotes the invasive behavior of colon cancer cells through modifying O-glycosylation of FGFR2
Source: Oncotarget. 2014 Mar 15;5(8):2096–106. doi: 10.18632/oncotarget.1815 (PMC4039148; doi:10.18632/oncotarget.1815)
Supplement: Supplementary file 1 [file oncotarget-05-2096-s001.pdf]

C1GALT1 overexpression promotes the invasive behavior of colon cancer cells through modifying O-glycosylation of FGFR2

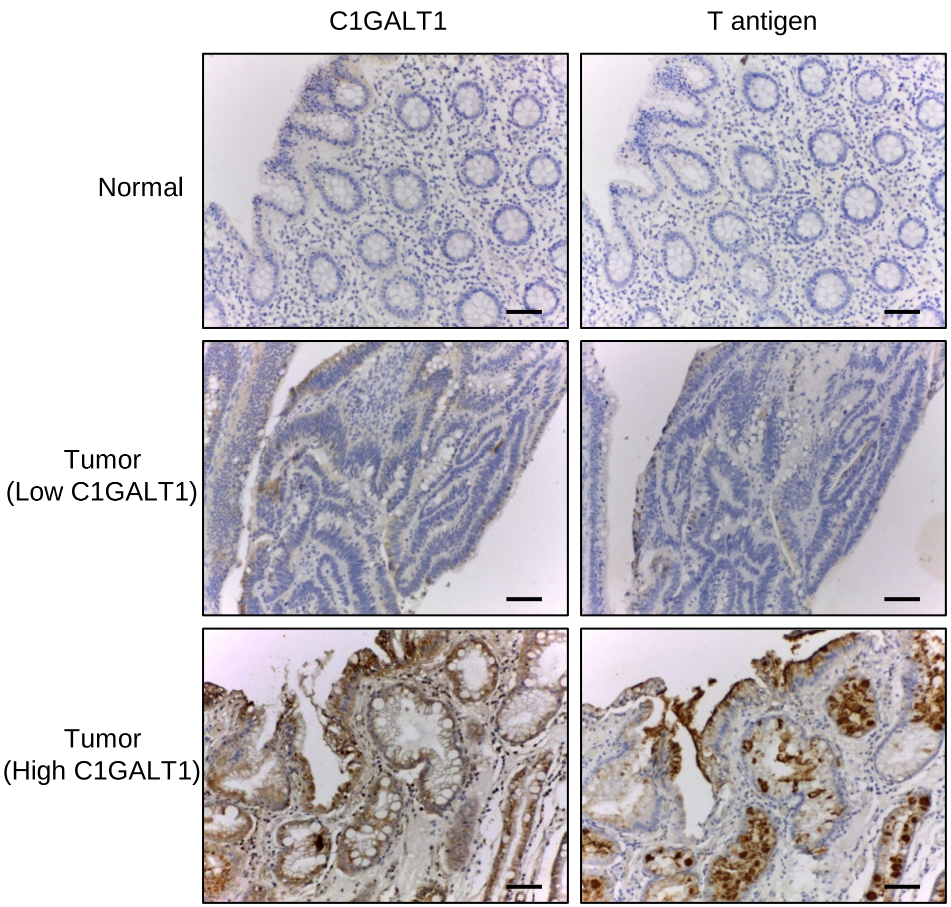

		T antigen		
		T > N	T ≤ N	Total
**C1GALT1**	T > N	29 (52.7%)	7 (12.7%)	36 (65.5%)
	T ≤ N	8 (14.5%)	11 (20.0%)	19 (34.5%)
Total		37 (67.2%)	18 (32.7%)	55 (100%)
				p = 0.004

Supplementary Figure S1: C1GALT1 expression is positively associated with T antigen expression in colorectal tumors. Representative images showing immunohistochemical staining of C1GALT1 and T antigen in serial sections of normal and tumor tissues (n = 55). Scale bars indicate 50 µm. T antigen was revealed by PNA staining. Statistical analyses indicate positive correlation between C1GALT1 and T antigen expression by *Kendall's* tau-b correlation test.

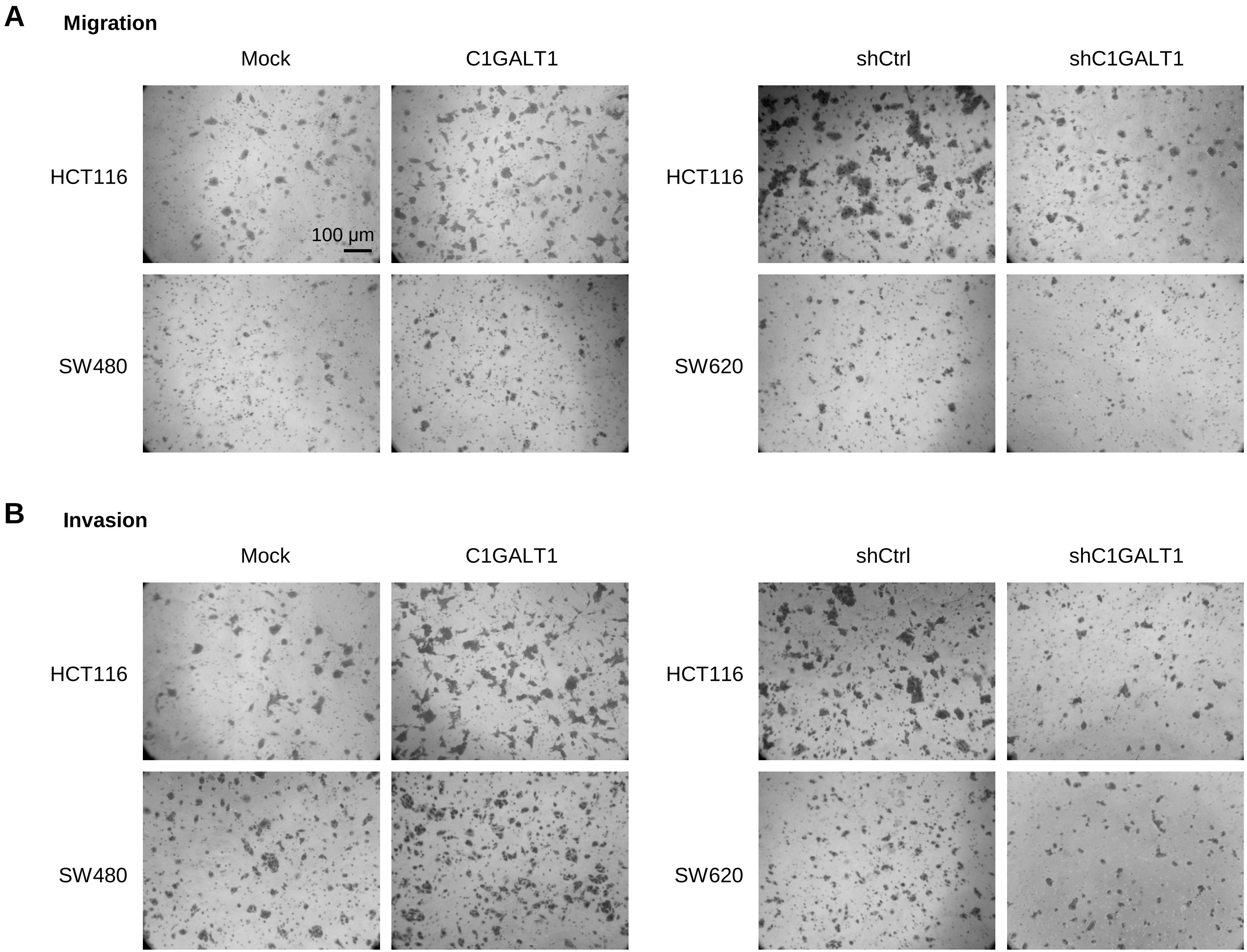

Supplementary Figure S2: Effects of C1GALT1 on migration and invasion. (A) Effects of C1GALT1 on colon cancer cell migration. (B). Effects of C1GALT1 on colon cancer cell invasion. Migrated and invaded cells were stained by crystal violet and photographed under a light microscope. The statistical results were shown in Figure 3B & 3C.

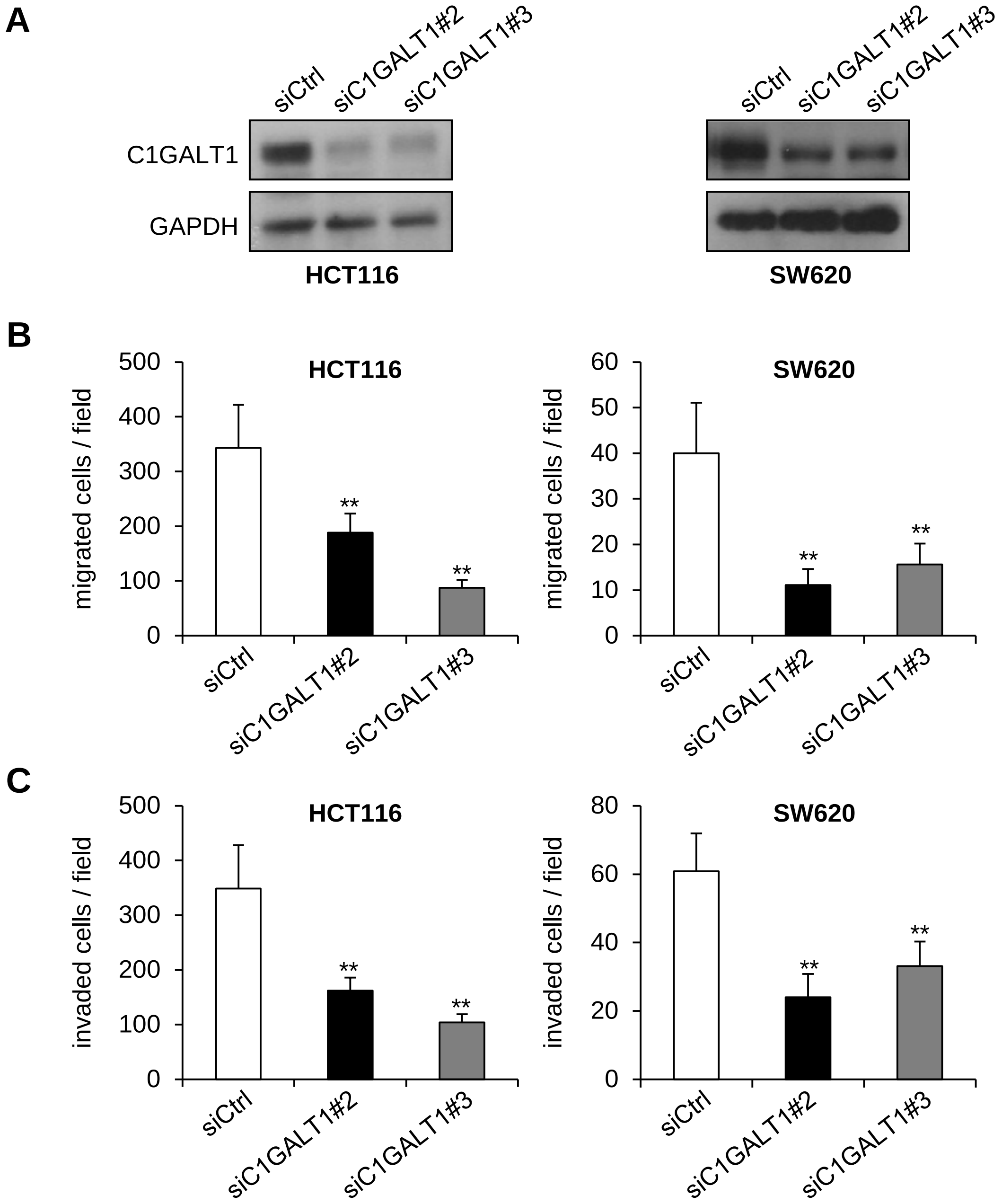

Supplementary Figure S3: Transient knockdown of C1GALT1 with siRNAs inhibits migration and invasion of colon cancer cells. (A) C1GALT1 knockdown with two different C1GALT1 siRNAs in HCT116 and SW620 cells. (B) C1GALT1 knockdown inhibits cell migration. (C) C1GALT1 knockdown inhibits cell invasion. Cell migration and invasion were analyzed by transwell migration assays and matrigel invasion assays, respectively. DMEM containing 10% FBS were used as chemoattractants and the number of migrated or invaded cells from 6 random fields was counted after 48 h. Results are presented as mean ± SD from three independent experiments. **$p < 0.01$.

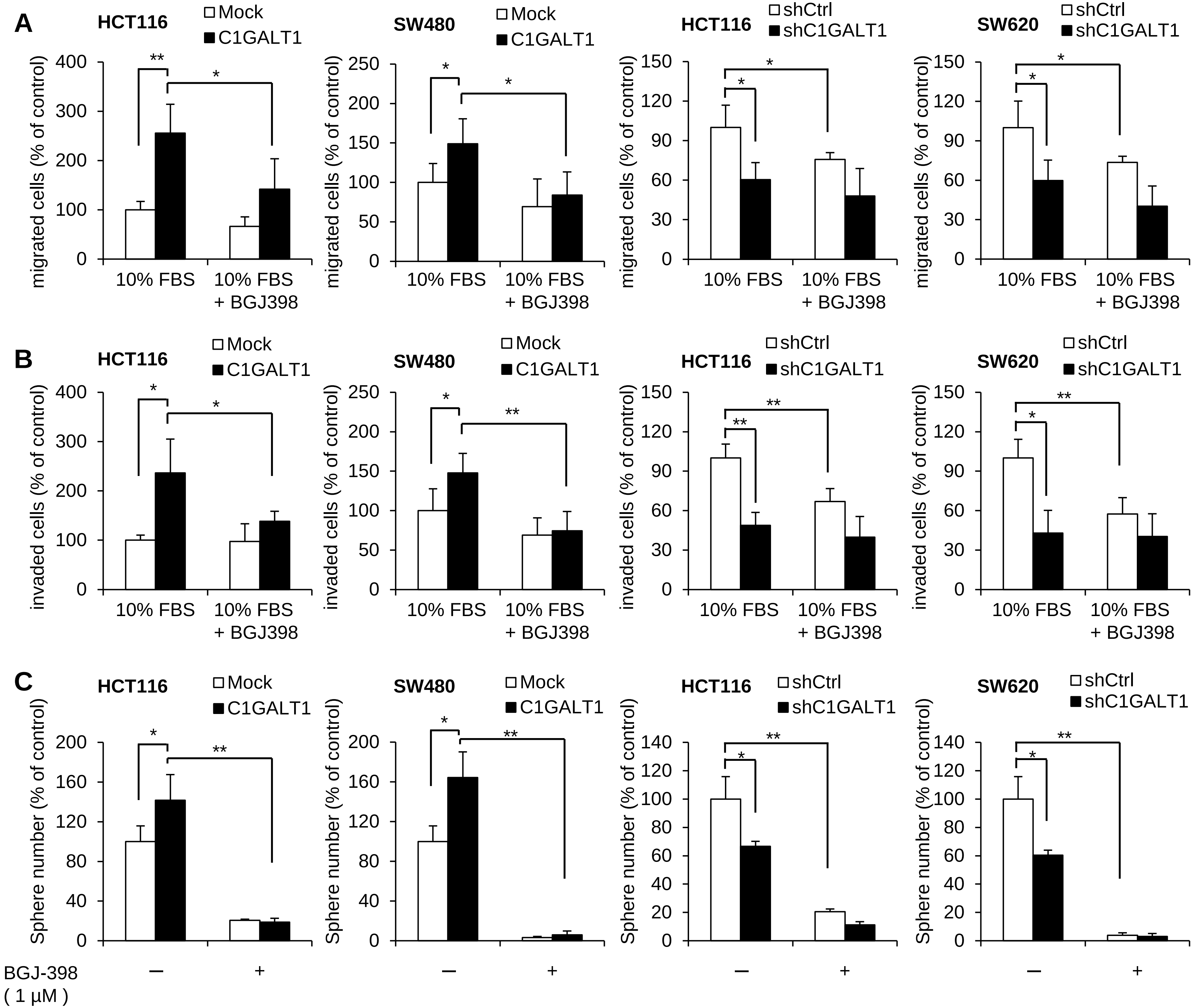
A
B
C
HCT116
SW480
SW620
Mock
C1GALT1
shCtrl
shC1GALT1
migrated cells (% of control)
invaded cells (% of control)
Sphere number (% of control)
10% FBS
10% FBS + BGJ398
BGJ-398 (1 μM)
−
+
*
**

Supplementary Figure S4: C1GALT1-enhanced migration, invasion and sphere formation are blocked by a FGFR inhibitor BGJ398. (A) Migration assays were performed to analyze effects of 1 µM BGJ398 on cell migration. DMEM containing 10% FBS were used as chemoattractants and the number of migrated cells was counted after 48 h. Results obtained are displayed as number of migrated cells and student's *t*-test expressing as mean ± SD, *$p < 0.05$. **$p < 0.01$. (B) Invasion assays were performed to analyze effects of 1 µM BGJ398 on cell invasion. DMEM containing 10% FBS were used as chemoattractants and the number of migrated cells was counted after 48 h. Results obtained are displayed as number of migrated cells and student's *t*-test expressing as mean ± SD, *$p < 0.05$. **$p < 0.01$. C, effects of 1 µM BGJ398 on C1GALT1-induced sphere formation. Sphere formation assays were performed in DMEM/F12 medium supplemented with 1 × B27, 20 µg/ml EGF and 25 µg/ml bFGF, with or without 1 µM BGJ398. Results obtained are displayed as relative number of spheres and student's *t*-test expressing as mean ± SD, *$p < 0.05$. **$p < 0.01$.

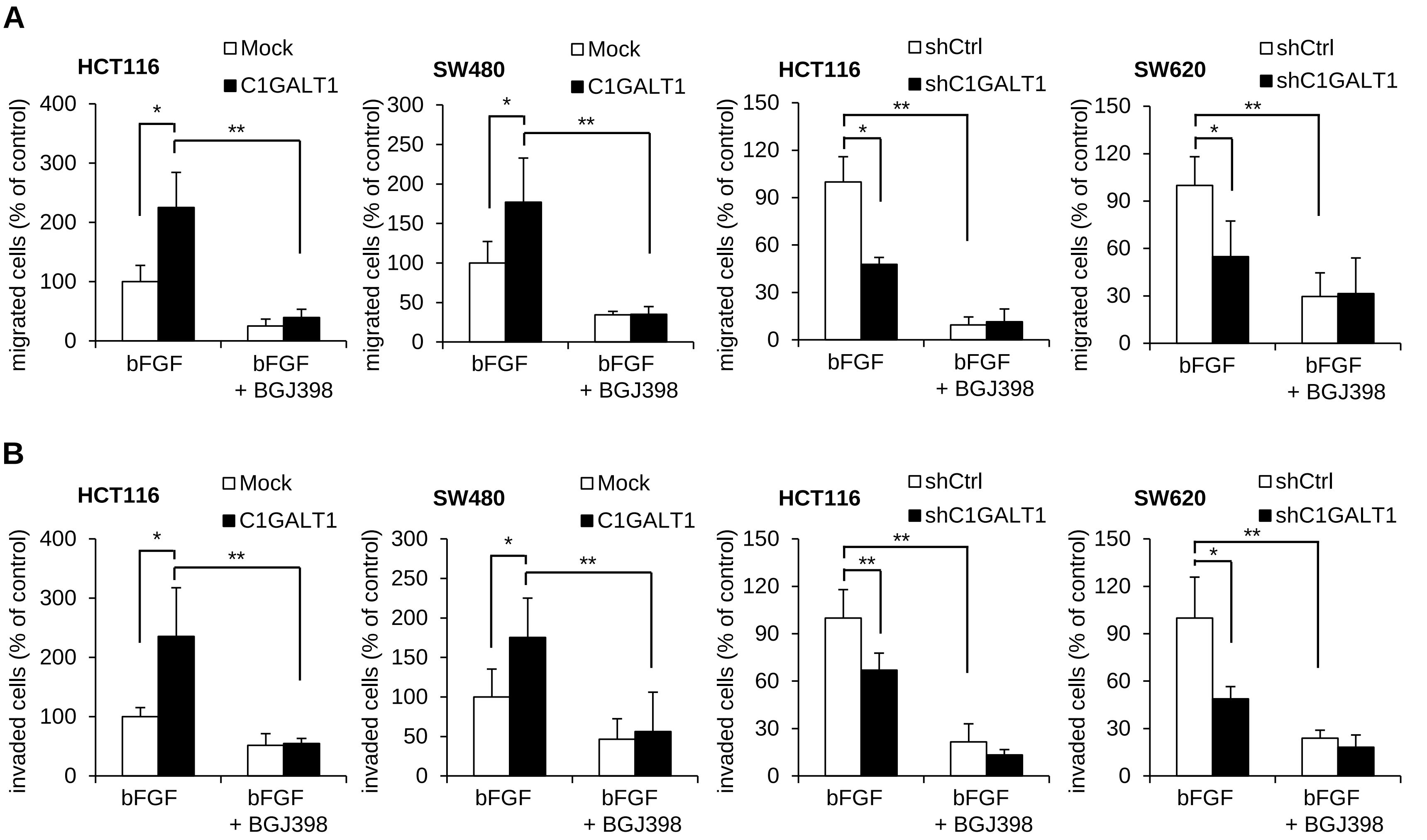

Supplementary Figure S5: BGJ398 inhibits the effects of C1GALT1 on bFGF-induced migration and invasion. (A) Migration assays were performed to analyze effects of 1 µM BGJ398 on bFGF-induced cell migration. 25 ng/mL bFGF were used as chemoattractants and the number of migrated cells was counted after 48 h. Results obtained are displayed as number of migrated cells and student's *t*-test expressing as mean ± SD, **p* < 0.05. ***p* < 0.01. (B) Invasion assays were performed to analyze effects of 1 µM BGJ398 on bFGF-induced cell invasion. 25 ng/ml bFGF were used as chemoattractants and the number of migrated cells was counted after 48 h. Results obtained are displayed as number of migrated cells and student's *t*-test expressing as mean ± SD, **p* < 0.05. ***p* < 0.01.

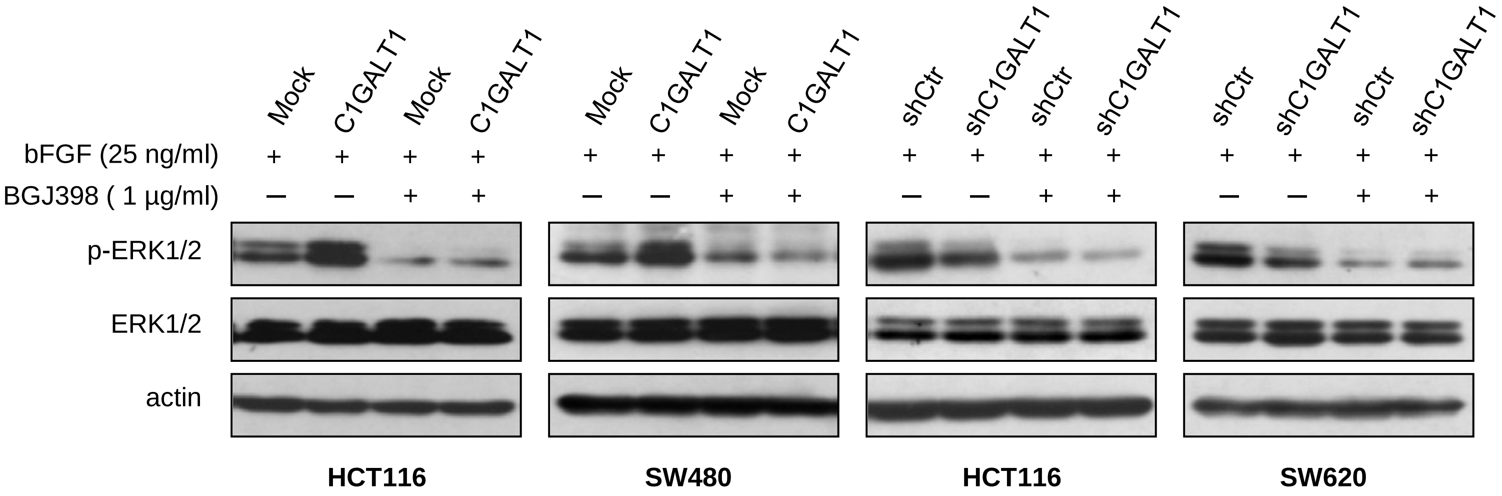

Supplementary Figure S6: BGJ398 decreases bFGF-induced phosphorylation of ERK1/2. Cells were serum starved for 24 h with or without 1 µM of BGJ398, and then treated with 25 ng/ml of bFGF. DMSO was used as control.
