# Supplementary material for: C1GALT1 overexpression promotes the invasive behavior of colon cancer cells through modifying O-glycosylation of FGFR2
Source: Oncotarget. 2014 Mar 15;5(8):2096–106. doi: 10.18632/oncotarget.1815 (PMC4039148; doi:10.18632/oncotarget.1815)
Supplement: Supplementary file 2 [file oncotarget-05-2096-s002.docx]

| Variable | No. of patients (%)  (n = 87) | | |
| --- | --- | --- | --- |
| Age(years) (mean$\pm\mathrm{SD}$) |  | | 66.5$\pm14.8$ |
| Gender |  | |  |
| Male |  | | 41 (47.1) |
| Female |  | | 46 (52.9) |
| Tumor location |  | |  |
| Colon |  | | 66 (76.7) |
| Rectum |  | | 20 (23.3) |
| Clinical stage |  | |  |
| I |  | | 16 (19.2) |
| II |  | | 20 (24.1) |
| III |  | | 25 (30.1) |
| IV |  | | 22 (26.5) |
| Histologic type |  | |  |
| Well to moderately differentiated |  | | 69 (93.2) |
| Poorly differentiated to undifferentiated | |  | 5 (6.8) |
| Distant Metastasis |  | | 25 (28.7) |
| Survival days |  | |  |
| Mean |  | | 1093.8 |
| Max |  | | 3769 |
| Min |  | | 52 |

Supplementary Table S1. Information of patients with colorectal cancer.
